# Microsatellite Development and First Population Size Estimates for the Groundwater Isopod *Proasellus walteri*

**DOI:** 10.1371/journal.pone.0076213

**Published:** 2013-09-27

**Authors:** Cécile Capderrey, Bernard Kaufmann, Pauline Jean, Florian Malard, Lara Konecny-Dupré, Tristan Lefébure, Christophe J. Douady

**Affiliations:** 1 UMR5023 Ecologie des Hydrosystèmes Naturels et Anthropisés, Université de Lyon, Université Lyon 1, Centre National de la Recherche Scientifique, Villeurbanne, France; 2 Institut Universitaire de France, Paris, France; Instituto de Higiene e Medicina Tropical, Portugal

## Abstract

Effective population size (*N*_e_) is one of the most important parameters in, ecology, evolutionary and conservation biology; however, few studies of *N*_e_ in surface freshwater organisms have been published to date. Even fewer studies have been carried out in groundwater organisms, although their evolution has long been considered to be particularly constrained by small *N*_e_. In this study, we estimated the contemporary effective population size of the obligate groundwater isopod: *Proaselluswalteri* (Chappuis, 1948). To this end, a genomic library was enriched for microsatellite motifs and sequenced using 454 GS-FLX technology. A total of 54,593 reads were assembled in 10,346 contigs or singlets, of which 245 contained candidate microsatellite sequences with suitable priming sites. Ninety-six loci were tested for amplification, polymorphism and multiplexing properties, of which seven were finally selected for *N*_e_ estimation. Linkage disequilibrium and approximate Bayesian computation methods revealed that *N*_e_ in this small interstitial groundwater isopod could reach large sizes (> 585 individuals). Our results suggest that environmental conditions in groundwater, while often referred to as extreme, are not necessarily associated with small *N*_e_.

## Introduction

Effective population size (*N*_e_) is one of the most important parameters in, ecology, evolutionary and conservation biology (e.g. [1-7]). *N*_e_ is classically defined as the number of breeding individuals in an idealized population (i.e. a panmictic population, with sex ratio of one and no overlapping generations) that has the same rate of change of allele frequencies or heterozygosity than the population under consideration [8]. Knowing *N*_e_ allows evaluation of the effects of genetic drift and natural selection, as well as their consequences for population viability in terms of genetic diversity loss or inbreeding effects (e.g. [2,9,10]). Accurate estimation of *N*_e_ has also become important from an analytical point of view since the formalization of the coalescent theory [11] and democratization of Bayesian inferences in population genetics, because informative priors significantly improve estimation and recovery of posterior densities (e.g. [12]). Last but not least, accessing *N*_e_ provides access to genetic parameters such as migration, mutation or recombination rates.

Despite community agreement about the importance of *N*_e_, it remains one of the most difficult parameters to assess in natural populations. First, no simple and general relationships exist between *N*_e_ and other demographic estimators such as total population size (population census size, *N*_c_ [5]) or density [13]. Indeed, the *N*_e_/*N*_c_ ratio does not only vary among taxa but is also likely to vary within species, especially in those showing high fecundity or high variation in density or reproductive success, such as arthropods and teleostean fishes [5,14]. Second, estimating *N*_e_ from genetic data is also a challenging task because various approaches capture different components of effective population size [5,15,16]. Estimations of contemporary (current) *N*_e_, the focus of this study by contrast to long-term Ne inferred from sequence-based phylogenetic methods integrating *N*_*e*_ over hundreds of generations, were initially based on temporal methods that analyzed several samples collected at different times from the same population [17-20]. These methods were time consuming and unmanageable for taxa displaying long generation times. However, recent analytical developments implemented single-sample approaches for estimating contemporary effective population size, using nuclear markers such as microsatellites and single nucleotide polymorphism [3,21,22]. As a consequence, estimates of contemporary *N*_e_, while still sparse, are becoming more frequent in the literature.

A survey of the current literature on freshwater metazoans revealed that most of our knowledge on *N*_e_ arises from studies of actinopterygian fishes, with a strong bias towards economically important Salmoniformes. In particular, much effort was devoted to highlight the effect of environmental variables [23] and anthropogenic disturbances such as dams [24] or restocking practices [25,26] on *N*_e_. Actinopterygian fishes hence provided a much-needed opportunity to compare approaches and methods to estimate *N*_e_ [7] or to infer the intraspecific variability of *N*_e_ (e.g. [27]). Aside from actinopterygian taxa, information is available for lampreys [28], aquatic snails [29,30], mussels [31], calanoid copepods [32] and crayfishes, in the latter for long-term *N*_e_ only [33,34]. Non-permanent aquatic dwellers did not receive much attention either with only a few studies dedicated to frogs [35,36], newts [37], hydrophilid beetles [38], caddisflies [39] and damselflies [40]. Yet, most studies dealt with surface water taxa from streams, lakes, marshes, and springs, despite the fact that a large number of aquatic phylogenetic lineages are restricted to or occur predominantly in groundwater [41]. Rare exceptions concerned the estimation of long-term *N*_e_ for groundwater crayfishes [33,42,43] and the estimation of *N*_e_ as a product of an unknown mutation rate for diving beetles [44].

In this study, we estimated the contemporary effective population size of the obligate groundwater isopod *Proaselluswalteri* (Chappuis, 1948). A genomic library was enriched for microsatellite motifs and sequenced using 454 GS-FLX technology. A total of 54,593 reads were assembled in 10,346 contigs or singlets, of which 245 contained candidate microsatellite sequences with suitable priming sites. Ninety-six loci were tested for amplification, polymorphism and multiplexing properties. Two genotyping multiplexes, for a total of seven loci were finally tested for cross-species transferability and used to infer contemporary effective population size in *P* *. walteri* .

## Materials and Methods

### Sampling and taxonomic identification

**Figure 1:**
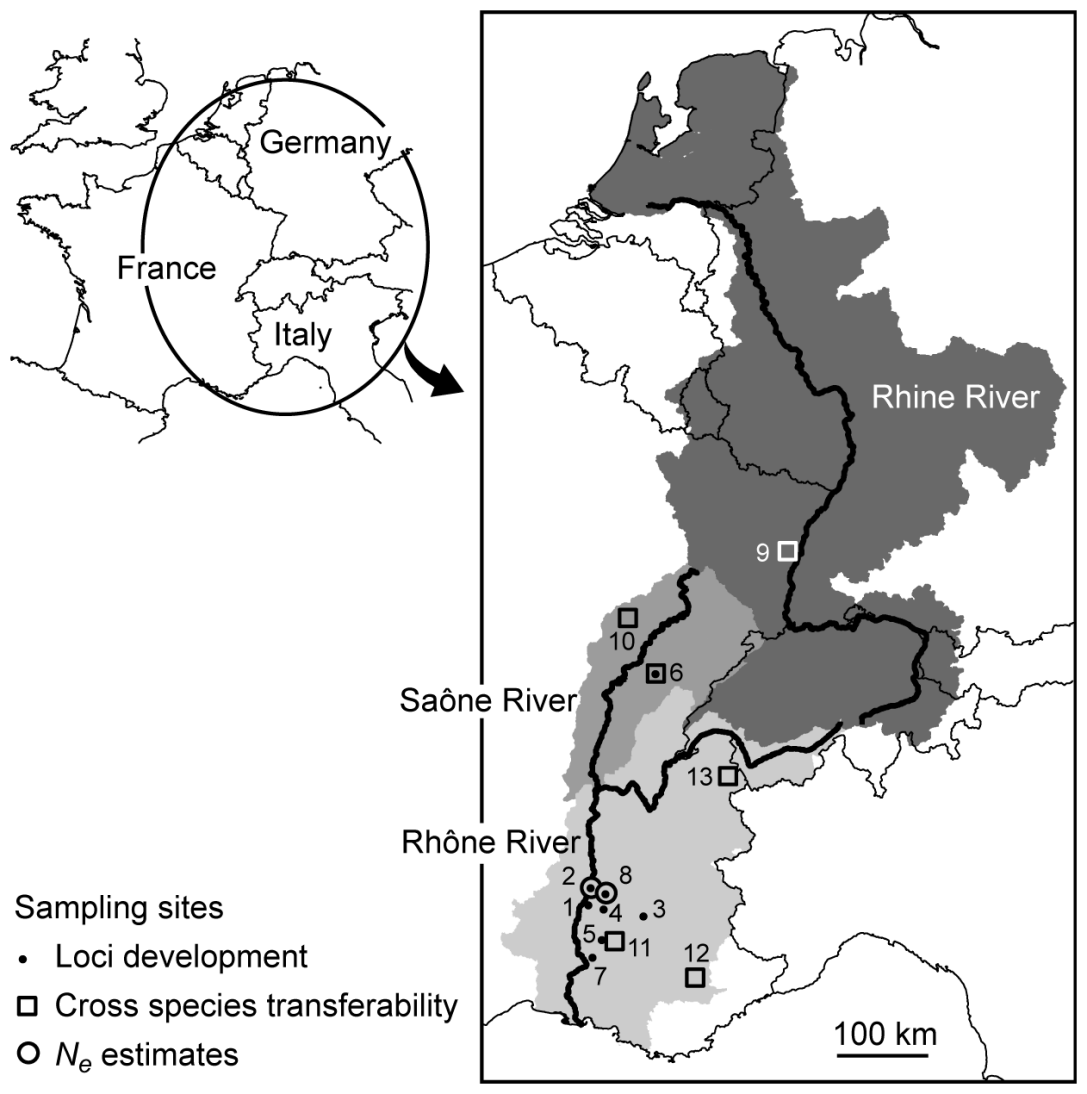
Map showing the location of sampling sites. Dark, light and very light grey patterns show the River catchments colonized by *Prosaelluswalteri* _T058, *P* *. walteri* _T059, and *P* *. walteri* _T060 (focal taxa), respectively. See Table 1 for site codes.

*P* *. walteri* morphospecies is a small (body length: 3 mm), eyeless, and depigmented isopod that inhabits the interstices in the hyporheic zone of rivers and groundwater in unconsolidated sediments. Its geographic range encompasses two major river catchments, the Rhine and Rhône Rivers (Figure 1). *Proasellus* is one of the most speciose genera in European groundwater, with approximately 120 species formally described in the literature. However, recent molecular studies by Morvan et al. [45] revealed that it contained a large number of unrecognized (morphologically cryptic) species. Morvan and coauthors distinguished three cryptic species within the *P* *. walteri* morphospecies: *P* *. walteri* _T058 is restricted to the Rhine River catchment, *P* *. walteri* _T059 to the Saône River catchment (a major tributary of the Rhône River) and *P* *. walteri* _T060 to the southern part of the Rhône River catchment (see Figure 1). Hereafter, the focal taxon in this study, *P* *. walteri* _T060, is referred to as *P* *. walteri* .

The microsatellite library was developed using *P* *. walteri* individuals from Sauzet site, six sites were used in amplification tests, five for polymorphism tests and two for *N*_e_ estimations (Figure 1; Table 1). Additionally, six species were collected to test for microsatellite loci cross-species transferability. Field sampling did not involve endangered or protected species and none of the sampling sites required specific permission.

**Table 1:** Description of sites and species, and their use in the study.

									Objective				
						Loci development					Cross-		Ne
Site name	River	Code	Longitude	Latitude	Species	Library	Amplif.	Polym.	HWE		transferability		estimates
Sauzet	Roubion	1	4.81867	44.59110	*P* *. walteri* _T060	**X**	**X**		**X**				
Livron	Drôme	2	4.84044	44.76574	*P* *. walteri* _T060		**X**	**X**	**X**				**X**
Les Prés	Drôme	3	5.62145	44.51219	*P* *. walteri* _T060		**X**	**X**					
Soyans	Roubion	4	5.03602	44.62615	*P* *. walteri* _T060		**X**	**X**					
Vaison la Romaine	Ouvèze	5	5.04771	44.24374	*P* *. walteri* _T060		**X**	**X**					
Belmont	Loue	6	5.59472	47.00945	*P* *. walteri* _T059		**X**	**X**			**X**		
Bédarrides	Ouvèze	7	4.92899	44.05605	*P* *. walteri* _T060				**X**				
Aouste	Drôme	8	5.05751	44.71454	*P* *. walteri* _T060				**X**				**X**
Rossfeld	Rhine	9	7.63116	48.33390	*P* *. walteri* _T058						**X**		
Crécey-sur-Tille	Tille	10	5.12611	47.56333	* P. strouhali *						**X**		
La Penne	Ouvèze	11	5.22722	44.24240	*P* *. synaselloides* _T084						**X**		
Senez	Asse	12	6.40837	43.91530	*P* *. synaselloides* _T085						**X**		
Salle	Giffre	13	6.75389	46.00139	* P. valdensis *						**X**		

Latitude and longitude are expressed in decimal degrees. Species names follow Morvan et al. (2013). Crosses indicate whether the corresponding sites were used for library construction, amplification tests, polymorphism tests, assessment of loci parameters (HWE), cross- transferability experiments and *N*_*e*_ estimates.

Individuals were collected using a Bou-Rouch pump [46] at a depth of 60 cm below the riverbed. After water elutriation, samples were placed in 96% ethanol at ambient temperature for transport back to the laboratory, then at 4°C until sorting and morphological identification. Specimens were identified at the species level using original species diagnoses, which are mostly based on the morphology of male copulatory organs (second pleopod). Given the prevalence of cryptic species in the *Proasellus* genus [45] and the occurrence of a morphologically similar species complex *Proasellussynaselloides* (Henry, 1963) living in sympatry with *P* *. walteri* , molecular verifications were conducted for each individual after genomic DNA extraction (see below). Following Chapman et al. [47], we developed a multiplex PCR scheme using one genus-specific forward primer (16S-ProaF1: 5’-CCTATGAGTCGTTTAAATGGCCGCA-3’ [48]) and two species-specific reverse primers (16S-Pwalt-R458: 5’- CTATCTATATATATATTTGCTTATATAGGG-3’, 16S-Psyna-R217: 5’- TAAAGTTTTATAGGGTCTTATCGTCCA-3’, for *P* *. walteri* and *P* *. synaselloides* complex, respectively) targeting the 16S mitochondrial DNA. Reverse primers were designed in such a way that PCR products were different in size (458 and 217 bp for *P* *. walteri* and *P* *. synaselloides* complex, respectively) and could be differentiated by electrophoresis on 1.5% agarose gels.

Molecular identifications of species selected for cross-species transferability of microsatellites (Table 1) were performed by sequencing a fragment of the 16S using genus-specific primers (Forward: as above; reverse 16Sbr: 5′- CCGGTCTGAACTCAGATCACGT -3’ [49]) and by comparing obtained sequences to available references 45. Standard PCRs were conducted with a final 15 µl volume containing 1.5 µl of standard buffer (10 X, Eurobio, Courtaboeuf, France), 0.24 µl of each primer (10 µM), 0.18 µl of MgCl_2_ (50 mM, Eurobio, Courtaboeuf, France), 0.15 µl of BSA (10 mg ml^-1^, New England Biolabs, Ipswich, USA), 0.13 µl of dNTP (20 mM, Eurogenetec), 0.13 µl of EurobioTaq DNA Polymerase (5 U µl^-1^, Eurobio, Courtaboeuf, France) and 2 µl of genomic DNA (*c.* 10 ng µl^-1^). Cycling conditions were as follows: (i) one step of initial denaturation (2 min) at 94°C, (ii) 36 cycles with denaturation (15 s) at 94°C, annealing (20 s) at 53 (16S-ProaF1/br) or 56°C (16S-ProaF1/16S-Pwalt-R458/-Psyna-R217), extension (30 s) at 72°C, and (iii) one step of final extension (5 min) at 72°C. Sanger sequencing was performed by Biofidal-Themis (Vaulx-en-Velin, France).

### Development of microsatellite markers

Genomic DNA was extracted using the chloroform extraction method optimized for *Proasellus* by Calvignac et al. [48]. Briefly, DNA was extracted from whole organisms following standard digestion (15 µg proteinase-K in 200 µl TNES buffer, 0.05 M Tris, 0.1 M NaCl, 0.01 M EDTA, 0.5% SDS) and salt-chloroform purification. DNA was resuspended in TE (TrisHCl, 10 mM, 1 mM EDTA) to a final volume of 16 µl. A microsatellite-enriched library was constructed by the Savannah River Ecology Laboratory according to Lance et al. [50] using a pooled DNA extract from 166 individuals of *P* *. walteri* collected at Sauzet site (Table 1). DNA was digested using RsaI, DNA fragments were ligated to SNX linkers, and dynabeads linked to di-, tri- and tetranucelotide microsatellite motives were used for enrichment. The enriched library was subsequently sequenced using a 454 FLX sequencer (454 Life Sciences/Roche Applied Biosystems, Nutley, NJ, USA). Raw DNA sequences were cleaned of remnant vectors and assembled in contigs with CAP3 [51]. Appropriate microsatellite motifs with suitable priming sites were subsequently identified using the program MSATCOMMANDER 1.0.4 [52]. Among these, 96 microsatellite loci were selected according to their type of motifs (di- and tetranucleotids), the length and purity of their repeat tract, and their size class. We favored loci with di- and tetranuclelotid motifs with the purest stretch and the most numerous repeats. Loci were also chosen so that three size classes (100-200 bp, 200-300 bp, and 300-400 bp) were kept in similar proportions to facilitate multiplexing. They were tested for amplification using *P* *. walteri* individuals from six sites (Table 1). For each site, DNA extracted from eight individuals was pooled because the limited amount of DNA recovered from single organisms (30 ng to 100 ng) would have been insufficient to carry out the 96 amplification tests. Next, polymorphism of amplifiable loci was evaluated using six non-pooled individuals from five sites (total number of individuals = 30, Table 1). PCR reactions were conducted using the Type-it TM Microsatellite PCR Kit (QIAGEN) in a 10-µl volume containing 5 µl of QIAGEN Multiplex PCR Master Mix, 3 µl of H_2_O, 1 µl of Primer Mix (2 µM), and 1 µl of DNA (20 ng µl^-1^). Cycling conditions were as follows: (i) one step of initial denaturation (5 min) at 95°C, (ii) 32 (amplification experiment) or 39 (polymorphism experiment) cycles with denaturation (30 s) at 95°C, annealing (90 s) at 57°C (amplification) or 50°C (polymorphism), extension (30 s) at 72°C, and (iii) one step of final extension (30 min) at 60°C. Amplification and polymorphism were tested on 1.5% or 3% agarose gels respectively.

### Microsatellite genotyping

Despite resolute efforts at optimization, only four loci could be combined into two PCR mixes of two loci and the three remaining loci had to be amplified separately (Table 2). PCRs for the two mixes of two loci (PCR mix 1: Pwalt_Di12 with Pwalt_Di21; and PCR mix 2: Pwalt_Te30 with Pwalt_Te39) were run using the Type-it TM Microsatellite PCR Kit (QIAGEN) in a 10-µl volume containing 5 µl of QIAGEN Multiplex PCR Master Mix, 1 µl of primer mix (F+R, 2 µM), 1µl of H_2_O, 1µl of QIAGEN Q-solution and 2 µl of genomic DNA (c. 10 ng µl^-1^). PCR reactions for Pwalt_Di31 and Pwalt_Di36 loci were run in a 10-µl volume containing 5.72 µl of H_2_O, 1 µl of standard buffer 10X (New England Biolabs, Ipswich, USA), 1 µl of primer mix (F+R, 2 µM), 0.1 µl of BSA (10 mg ml^-1^, New England Biolabs, Ipswich, USA), 0.09 µl of dNTP (20 mM, Eurogenetec), 0.09 µl of Taq DNA Polymerase (5 U µl^-1^, New England Biolabs, Ipswich, USA) and 2 µl of genomic DNA (c. 10 ng µl^-1^). PCRs for Pwalt_Te46 locus were conducted in a 10-µl volume containing 5.32 µl of H_2_O, 1 µl of standard buffer (10 X, Eurobio, Courtaboeuf, France), 1 µl of primer mix (2 µM), 0.4 µl of MgCl_2_ (50 mM, Eurobio, Courtaboeuf, France), 0.1 µl of BSA (10 mg ml^-1^, New England Biolabs, Ipswich, USA), 0.09 µl of dNTP (20 mM, Eurogenetec), 0.09 µl of EurobioTaq DNA Polymerase (5 U µl^-1^, Eurobio, Courtaboeuf, France) and 2 µl of genomic DNA (c. 10 ng µl^-1^). Cycling condition were set as follows: (i) one step of initial denaturation (5 min) at 95°C (both mixes) or 94°C (3 independent PCRs); (ii) 39 cycles with denaturation (30 s) at 95°C (both mixes) or 94°C (3 independent PCRs), annealing for 90 s (both mixes) or 30 s (3 independent PCRs) at 58°C (Mix1) or 56°C (Pwalt_Di31 locus) or 54°C (Mix3 and Pwalt_Di36 locus) or 48°C (Pwalt_Te46 locus) and extension (30 s) at 72°C; and (iii) one step of final extension at 60°C for 30 min (both mixes) or 72°C for 10 min (3 independent PCR). PCR products were diluted 50 times and pooled into two genotyping mixes: 1) genotyping mix A containing PCR mixes 1 and 2, and 2) genotyping mix B containing the three loci amplified separately (Table 2). These mixes were then beads-purified (AxyPrep Mag PCR Clean-up, Axygen, Union City, CA, U.S.A.) and genotyped by a service provider (Biofidal-Themis, Vaulx-en-Velin, France) on a 3730xl DNA Analyzer (Applied Biosystems).

**Table 2:** Seven microsatellite loci from *P* *. walteri* with repeat motifs, PCR primers, fluoro-label marking, size ranges in base pairs (bp), PCR annealing temperatures (*T*_*a*_) and Genbank accession numbers.

	Locus	Repeat motif	Primer sequence (5' -3')	Fluoro-label	Alelle size range (bp)	*T*_*a*_ (°C)	GenBank accession number
**Genotyping mix A**	*PCR mix 1*						
	Pwalt_Di12	(AG)_12_	F: CGGAGTGGTGTGTGAAATCTTC	**NED**	89-209	58	KF423438
			R: TTCCAGGCAGAACGAATTGC				
	Pwalt_Di21	(AG)_12_	F: AACGTCGAATACCCACTCAGAG	**HEX**	99-143	58	KF423439
			R: TCTCTCTAAGTGGATCGGCAAG				
	*PCR mix 2*						
	Pwalt_Te30	(ACAG)_8_	F: AAATTGACAAAGTCCAGTTCCG	**HEX**	190-258	54	KF423440
			R: ATTCTGCTTCTTTATTCCATCGTG				
	Pwalt_Te39	(ACTC)_9_	F: GAGCTGAACAACTACTGGCTTC	**6-FAM**	218-298	54	KF423441
			R: GAAGTTATTCGTCGTCAGCTCC				
**Genotyping mix B**	*Simplex*						
	Pwalt_Di31	(AG)_12_	F: TTCGTTGCAGAGACGATGAATG	**PET**	152-192	56	KF423442
			R: CGTAAGGTCTCTGTGAAGTCTTC				
	*Simplex*						
	Pwalt_Di36	(AG)_12_	F: GTTGGTGTTTGCTGCAACTC	**HEX**	162-182	54	KF423443
			R: TACTGCACTGCCGCTATACAAG				
	*Simplex*						
	Pwalt_Te46	(ACTC)_11_	F: ACATTTGTTCTTTGGTGGAGGC	**HEX**	300-410	48	KF423444
			R: TGCTAAATTTCATCGTTCTAGCATC				

For each locus, alleles were scored independently by two operators with GENEMARKER v.1.95 (SoftGenetics), using GS600 LIZ size standard (Applied Biosystems). Every ambiguous genotype was amplified and genotyped twice. The presence of null alleles or allelic dropout was tested using MICRO-CHECKER [53]; when a significant presence was detected (p<0.05), null alleles frequencies were calculated. Heterozygosity was measured at four sites (Livron, Aouste, Sauzet, and Bédarrides) using GENETIX v.4.0.5.2 [54]. Deviation from Hardy-Weinberg equilibrium (HWE) and linkage disequilibrium (LD) were estimated at the same sites using GENEPOP v.4.0 [55,56]. Significance values were adjusted for multiple testing (HWE) and comparisons (LD) using sequential Bonferroni corrections [57].

### Cross species transferability

Assessing microsatellite transferability for all *Proasellus* species was far beyond the scope of this paper. Instead, we selected one site for each of the two other cryptic species within *P* *. walteri* morphospecies, one site for *P* *. strouhali* , which is the sister species of *P* *. walteri* complex, one site for the more distantly related morphospecies *P* *. valdensis* and two sites for *P* *. valdensis* sister group, the *P* *. synaselloides* complex (Table 1 [45]).

### Estimation of effective population size

We estimated effective population size for two sampling sites (Table 1) using LDNe [22] and ONeSAMP [21]. LDNe uses a linkage disequilibrium method initially developed by Hill [58] and corrected for small sample size [59] to estimate *N*_e_ from a single population sample, whereas ONeSAMP relies on approximate Bayesian computation (ABC). For LDNe, the lowest allele frequency used was 0.02. As no prior information was available on *P* *. walteri* census or effective population sizes, we used two as the lower value and 10,000 as the upper value for priors on *N*_e_ in ONeSAMP. Three exact replicate runs per dataset were performed as cross validation tests on the online ONeSAMP. As neither LDNe nor ONeSAMP should be used with loci showing LD [21,22], calculations were made dropping one locus per pair in LD, to strictly comply with the conditions imposed by the methods.

## Results

### Microsatellite characterization

A total of 54,593 reads were obtained using 454 sequencing from the enriched library of genomic DNA from *P* *. walteri* . Mean read length reached 242 bp for 13.10^6^ bp of total sequence length. CAP3 assembled those reads in 4,225 contigs leaving 6,121 reads as singletons. Raw data were deposited in EBI ENA database (http://www.ebi.ac.uk/ena/) under the study accession number PRJEB4363. MSATCOMMANDER identified 4,518 sequences that contained di-, tri- or tetranucleotid microsatellite loci (File S1), but it was able to define amplification primers for only 245 microsatellite loci (File S2). Among these, 96 loci were retained according to selection criteria. A total of 32 loci produced reliable amplification (i.e. clear band at expected size) in at least 3 out of 6 sampling sites (Table 1, Table S1) and 19 were found to be polymorphic (i.e. harbored multiple bands) on agarose gels. After fluorescent labeling, seven of these 19 loci were found to produce a clear signal without parasite peaks and at expected size, a strong signal to noise ratio and limited stutters. Thus, a total of 7 loci were considered to provide reliable genotyping (Table S1).

Gene diversity indices are reported in Table 3. Total number of alleles per locus was 11 in Pwalt_Di36, 15 in Pwalt_Di21 and Pwalt_Te30, 20 in Pwalt_Te39, 21 in Pwalt_Di31, 31 in Pwalt_Di12 and 42 for Pwalt_Te46 for a total 155 alleles. The number of alleles per locus per site ranged from five to 28 in 30 individuals genotyped at Sauzet and Bédarrides, and from seven to 30 in 90 individuals at Aouste and Livron. All loci had private alleles in at least one site and three loci had private alleles in all sites (Pwalt_Di12, Pwalt_Di21 and Pwalt_Te46). Importantly, the number of alleles and private alleles correspond to minimal values since homoplasy and point mutations in both flanking regions and microsatellite motives were not assessed. Observed and expected heterozygosities ranged from 0.300 to 0.933 and 0.362 to 0.918, respectively. Significant deviations from HWE were found in three loci out of seven and in two sites (Pwalt_Te39 in Livron and Aouste, Pwalt_Di12 in Aouste and Pwalt_Di21 also in Aouste). Tests with the program MICRO-CHECKER indicated significant null allele presence in three out of four cases of significant HWE deviations (Table 3), sometimes at high frequency (e.g. 0.196 and 0.166 for Pwalt_Te39 in Aouste and Livron, respectively). After applying sequential Bonferroni corrections no LD was detected over all four sites (*p* > 0.573). However, within sites, Pwalt_Te30 and Pwalt_Te39 were in LD in Livron (*p* = 0.014). As a consequence, *N*_e_ was estimated in Livron without Pwalt_Te39.

**Table 3:** Summary data for microsatellites developed for *P* *. walteri* .

Locus	Sites name	Rivers	N	A	pA	Ho	He	F	*p* HWE	NA
**Pwalt_Di12**										
	Livron	Drôme	90	21	4	0.7556	0.8048	0,061	**0.0321^b^**	
	Aouste	Drôme	88	24	6	0.7500	0.7972	0,059	**0.0000^a^**	
	Sauzet	Roubion	30	10	2	0.8000	0.7994	-0,001	0.4347	
	Bédarrides	Ouvèze	30	7	1	0.6000	0.6867	0,126	0.1158	
**Pwalt_Di21**										
	Livron	Drôme	90	9	2	0.7778	0.8713	0,107	**0.0124^b^**	0.0544
	Aouste	Drôme	90	11	1	0.7778	0.8602	0,096	**0.0082^a^**	0.0477
	Sauzet	Roubion	30	5	1	0.3000	0.3622	0,172	0.1121	
	Bédarrides	Ouvèze	30	8	2	0.6333	0.6422	0,014	0.5021	
**Pwalt_Di31**										
	Livron	Drôme	90	17		0.8889	0.8948	0,007	**0.0340^b^**	
	Aouste	Drôme	90	19	2	0.9111	0.9048	-0,007	0.5409	
	Sauzet	Roubion	29	5	1	0.6552	0.6790	0,035	0.4603	
	Bédarrides	Ouvèze	30	11		0.9333	0.8139	-0,147	0.9915	
**Pwalt_Di36**										
	Livron	Drôme	90	7		0.7667	0.7377	-0,039	0.7526	
	Aouste	Drôme	90	7		0.7000	0.7457	0,061	0.3380	
	Sauzet	Roubion	29	10	1	0.8966	0.8454	-0,060	0.8356	
	Bédarrides	Ouvèze	30	9	1	0.8667	0.7961	-0,089	0.8840	
**Pwalt_Te30**										
	Livron	Drôme	90	12		0.7778	0.7717	-0,008	0.5369	
	Aouste	Drôme	90	13		0.7889	0.7983	0,012	0.6276	
	Sauzet	Roubion	30	12	1	0.7667	0.8222	0,068	0.1060	
	Bédarrides	Ouvèze	29	10		0.6552	0.8228	0,204	**0.0119^b^**	0.1071
**Pwalt_Te39**										
	Livron	Drôme	86	12		0.4302	0.6627	0,351	**0.0000^a^**	0.1662
	Aouste	Drôme	87	14	4	0.4713	0.7862	0,401	**0.0000^a^**	0.196
	Sauzet	Roubion	30	11	1	0.9000	0.8567	-0,051	0.7382	
	Bédarrides	Ouvèze	30	8		0.8333	0.7772	-0,072	0.5078	
**Pwalt_Te46**										
	Livron	Drôme	87	30	5	0.8851	0.9181	0,037	0.4053	
	Aouste	Drôme	89	28	5	0.9326	0.9111	-0,024	**0.0374^b^**	
	Sauzet	Roubion	29	8	2	0.7931	0.7658	-0,036	**0.0451^b^**	
	Bédarrides	Ouvèze	28	28	4	0.7857	0.8246	0,047	0.3664	

Number of individual (N), Number of alleles (A), private allelic richness (pA), observed (H_*O*_) and expected (He) heterozygosities, fixation index (*F*), probability of deviation from Hardy-Weinberg equilibrium (*p* HWE) and frequency of null alleles (NA) are given for each locus for N individuals from four sampling sites of *P* *. walteri* . Uncorrected significant deviations from HWE are in bold. Frequencies of null alleles are given only when the probability of their presence was significant (*p* < 0.05)

^a^ Significant after sequential Bonferroni correction; ^b^ Not significant after sequential Bonferroni correction

### Microsatellite transferability

Cross-species amplification success rates were highly variable among species (Table 4). The seven loci were found to amplify better in *P* *. walteri* ’s closest relatives as both *P* *. walteri* _T058 and *P* *. walteri* _T059 were successfully amplified for all loci in at least three out of five individuals and amplifications gave polymorphic products for all but one locus per species (Pwalt_D31 in *P* *. walteri* _T058). Cross amplification in *P* *. strouhali* failed for four loci and produced monophorphic fragments for the three remaining loci. In constrast, three loci were polymorphic in *P* *. synaselloides* complex and four in *P* *. valdensis* . Cross-amplifications were also highly variable among loci. Products were obtained from all species for Pwalt_Di12 and Pwalt_Di21 loci (86 and 73% of individuals, respectively), whereas Pwalt_Di36 gave reliable products solely in the *P* *. walteri* complex.

**Table 4:** Cross-species amplification tests showing amplicon size ranges for the seven microsatellite loci in six different *Proasellus* species.

Species	Sites name	Pwalt_Te30	Pwalt_Te39	Pwalt_Di12	Pwalt_Di21	Pwalt_Di31	Pwalt_Di36	Pwalt_Te46
*P* *. walteri* _T058	Rossfeld	218-266 (5)	178-278 (4)	87-101 (5)	99-103 (5)	150 (5)	164-172 (5)	306-330 (3)
*P* *. walteri* _T059	Belmont	206-222 (4)	198-214 (4)	93-211 (5)	101-105 (5)	156-186 (4)	158-164 (5)	310-338 (3)
* P. strouhali *	Crécey-sur-Tille	—	178 (5)	111 (5)	113 (1)	—	—	—
*P* *. synaselloides* _T084	Lapenne	206 (2)	178 (1)	89-115 (5)	99-111 (5)	144-158 (2)	• (2)	—
*P* *. synaselloides* _T085	Senez	210 (1)	—	93-103 (2)	101-111 (2)	176 (1)	• (1)	294-300 (1)
* P. valdensis *	Salles	—	178-290 (5)	87-179 (4)	93-101 (4)	116-184 (2)	• (2)	—

Numbers between brackets indicate the number of individuals (out of five individuals) with positive amplification, • indicates non-specific amplification, — indicates negative amplification in all individuals.

### Effective population size

*N*_e_ point estimates and associated 95% confidence intervals are reported in Table 5. LDNe provided large estimates of *N*_e_ in Livron (*N*_e_ = 892) and Aouste, where a negative value indicated that *N*_e_ was indistinguishable from infinity [60]. The lower confidence interval in Livron was 243 and reached 585 in Aouste. Upper confidence intervals reached infinity in both estimates. ONeSAMP results proved more challenging to analyze, as replicate runs did not seem to converge in Aouste, with point estimates ranging from 533 to 1844. Replicate runs were more stable in Livron where point estimates ranged from 142 to 186, with lowest and highest confidence intervals of 83 and 327, respectively. LDNe point estimates were hence always higher than those provided by ONeSAMP, and in Livron, the LDNe estimate was even far above the highest confidence interval provided by ONeSAMP (892 vs 327).

**Table 5:** Estimated mean effective population size (*N*_*e*_) of *P* *. walteri* at two river sites inferred from 90 individuals using LDNe and ONeSAMP.

		Livron 6 loci (without Pwalt_Te39)	Aouste 7 loci
*N*_e_ - LDNe		892 (243 - Infinite)	-1470 (585 - Infinite)
*N*_e_ - ONeSAMP	Run 1	186 (103-327)	533 (202-1995)
	Run 2	182 (93-314)	1209 (434-5855)
	Run 3	142 (83-260)	1844 (557-10265)

Brackets show 95% confidence intervals

## Discussion

We successfully identified 4,518 microsatellite loci in the non-model organism *P* *. walteri* . Of these, 245 contained flanking regions sufficiently long for further amplification, 96 were tested and seven proved to be useful for subsequent analyses in population genetics. This demonstrates that despite tremendous advances made over recent years in the development of microsatellite markers, this process can still be a challenging endeavor. Library screening using high throughput sequencing facilities can now be performed even for non-model organisms such as *P* *. walteri* and can reveal a large number of microsatellite loci. However this first stage, while being crucial to start with a large number of candidates, is by no means a guarantee as the number of useful loci may dwindle dramatically along the development process.

Our finding that sampling sites were characterized by highly polymorphic microsatellite loci does not support prevailing views that populations of subterranean organisms do not support high genetic diversity (e.g. [61,62]). While surprising at the site level, high diversity among sites, as is evidenced by numerous private alleles, was somewhat more expected. In a recent diversification study of the Aselloidea, Morvan et al. [45] suggested using a Bayesian relaxed clock model that the three cryptic species of the *P* *. walteri* complex diverged between six and 10 million years ago. Given this time frame, populations of *P* *. walteri* _T060 sampled in this study might have diverged a long time ago, thereby providing a reasonable explanation for both the high among-sites allelic diversity and the significant frequency of null alleles encountered at some sites and loci. Linkage disequilibrium between loci was found in one site only. Therefore, linkage processes affecting these loci, while highly significant were probably not caused by physical proximity on chromosomes. However, none of the many other processes resulting in linkage disequilibrium, such as bottlenecks, population substructuring or local selection regimes [63] could be dismissed here.

Cross-species amplification was successful and polymorphism was high among species of the *P* *. walteri* complex, but both were hard to predict from phylogenetic distances in more distantly related species. Indeed, *P* *. strouhali* is more closely related to *P* *. walteri* complex than any of the other three species considered in this study [45]. Yet, cross amplifications in *P* *. strouhali* failed or were uninformative for all loci, whereas three and four loci were polymorphic in *P* *. synaselloides* complex (i.e. two cryptic species) and *P* *. valdensis* , respectively. The expectation that cross-species amplification and polymorphism decrease with increasing genetic distance between the species from which the loci were isolated and the target species was validated in several vertebrate taxa [64,65]. It is however poorly documented in arthropods, and even much less so in freshwater taxa. Primmer et al. [64] suggested that the negative relationship between transferability and genetic distance was linear while Carreras-Carbonell et al. [65] argued it was logarithmic. Our results, if confirmed by additional cross-species experiments, are more compatible with the pattern proposed by Carreras-Carbonell et al. [65], with a high success rate between very closely related species (i.e. within *P* *. walteri* morphospecies) followed by a sharp decrease and large variance as phylogenetic distance increases. Whatever the relationship, success rates in cross species amplification and polymorphism reported in the present study suggest that the loci we characterized and developed might be of significant interest for future studies on the ecology of species within the *P* *. walteri* complex. These species are widespread in the hyporheic zones of two major catchments (the Rhine and Rhône rivers) that experienced contrasted Pleistocene climates, thereby providing a rare opportunity to test for the effect of past environmental conditions on population dynamics.

Estimates in *P* *. walteri* pointed towards large *N*_*e*_, with a point estimate of 892 in Livron and a minimum of 585 individuals in Aouste. While similar to *N*_*e*_ estimated in two species of calanoid copepods (*N*_e_ = 672.7 and 1027.4 [32]), these estimates are at odds with the small sizes inferred in snails (*N*_e_ ≤ 10 [29]; or *N*_e_ ≤ 245 [30]), damselflies (*N*_e_ ≤ 250 in most sites and for most methods [40]) or hydrophilid beetles (*N*_e_ < 20 [38]). There was however a marked difference between the two sites investigated, as in Aouste, a very large *N*_*e*_ seemed to prevent LDNe from providing a point estimate different from infinity, and resulted in the absence of convergence between ONeSAMP runs [5]. In contrast, ONeSAMP runs returned congruent values of *N*_*e*_ in Livron and both approaches suggested a smaller *N*_e_ than in Aouste. Such variations are consistent with current literature that often report *N*_*e*_ estimates varying by several orders of magnitude in the same species (e.g. [30,36]). Furthermore, our observation empirically corroborates simulations suggesting that accurate and precise estimations can hardly be achieved for moderate or large *N*_*e*_ (> c. 500[60]), even when large amounts of genetic information are available. Our inferences rely on large sample sizes (>87 individuals per population) and high allelic richness (averages of 15.4 and 16.6 for Livron and Aouste, respectively), but on seven loci only. However, as stated by Luikart et al. [5], “precision for estimates of Ne can be improved by roughly the same amount by sampling more individuals or by sampling more microsatellite loci” [18,60,66,67]. We used approximately 3 to 4 time more individuals per population than in most studies (e.g. [25,29,30,36,39]). Moreover, allelic richness of our loci was high as compared to other studies of freshwater organisms (e.g. [30,35,39]). A high allelic richness should theoretically increase the precision of Ne estimates by LDNe as well as that of ONeSAMP summary statistics (e.g. for He [68]). LDNe estimates rely on the number of independent comparisons: (n_i,j_ = (k_i_ - 1) * (k_j_ - 1), where k_i_ and k_j_ represent the number of alleles at loci i and j, respectively [10]). Altogether, this suggests that obtaining precise estimations for moderate to large N_*e*_ probably requires fulfilling the following conditions: improving estimation methods, acquiring many more loci using techniques such as Rad-tag sequencing [69] and getting even larger sample sizes [5].

Importantly, our inference of large *N*_*e*_ in an obligate groundwater organism such as *P* *. walteri* may not be as surprising as prevailing views may suggest. Indeed, Kane et al. [70] using allozymes heterozygosity, and a calculation based on the classical relation He=4N _e_µ/(4N _e_µ +1) where He is the expected heterozygosity and µ the mutation rate, also reported large *N*_*e*_ for *Gammarus* *minus* amphipods (*N*_e_ = 2x10^5^) and *Astyanax* *fasciatus* teleosteans (*N*_e_ = 9x10^4^). For comparison, using the same formula with our data, He for the least and most polymorphic loci, and a mutation rate of 10^-4^ for microsatellites, returned slightly lower *N*_*e*_ ranges of 5x10^3^ to 3x10^4^ in Livron and 9x10^3^ to 3x10^4^ in Aouste. However, calculations based on this formula provide rough estimates that cannot be compared with LDNe or ONeSAMP estimates. Finding large *N*_e_ in groundwater taxa suggests that environmental factors in groundwater, while often referred to as extreme, are not necessarily associated with small *N*_*e*_. However, our results desperately call for further assessments of *N*_*e*_ in groundwater as well as in closely related surface-dwelling species. Such comparisons will make it possible to formally test another central question in groundwater ecology: is *N*_*e*_ reduced in groundwater-dwellers when compared to their surface counterparts? To our knowledge, the best evidence in favor of decreased *N*_*e*_ in groundwater comes from mitochondrial comparisons in crayfishes [33]. However, Buhay & Crandal [33] documented changes in long-term *N*_e_ of crayfishes rather than changes in contemporary *N*_e_. Other evidences come from reduced heterozygosity values in groundwater [61,62,71], but this reduction may not be systematic [72] nor readily interpretable in terms of *N*_e_ [73]. Importantly, to solve this issue, alternative molecular methods such as Single Nucleotide Polymorphism detection and calling using RAD-tag sequencing [69] seem very promising as they may provide thousands of loci when it proves difficult to develop more than tens loci in most, including ours, microsatellite studies. These methods will also make it possible to access other long-standing issues including spatial delineation of population and dispersal processes.

## Supporting Information

File S1 **4,518 sequences identified by MSATCOMMANDER containing di-, tri- or tetranucleotid microsatellite loci.**
(TXT)

File S2 **245 microsatellite loci identified by MSATCOMMANDER with suitable priming regions.**
(TXT)

Table S1 **96 loci selected using the nature, purity and number of repetitions as criteria and tested for the development of microsatellite markers in *P* *. walteri* .**
(XLS)
